# Chemical Characterization and Antifungal Activity of Blue Tansy (*Tanacetum annuum*) Essential Oil and Crude Extracts against *Fusarium oxysporum* f. sp. *albedinis,* an Agent Causing Bayoud Disease of Date Palm

**DOI:** 10.3390/antibiotics12091451

**Published:** 2023-09-16

**Authors:** Hajar Ettakifi, Kaoutar Abbassi, Safae Maouni, El Hadi Erbiai, Abderrahmane Rahmouni, Mounir Legssyer, Rabah Saidi, Zouhaire Lamrani, Joaquim C. G. Esteves da Silva, Eugénia Pinto, Abdelfettah Maouni

**Affiliations:** 1 Biology, Environment, and Sustainable Development Laboratory, Higher School of Teachers (ENS), Abdelmalek Essaadi University, Tetouan 93000, Morocco; hajar.ettakifi@etu.uae.ac.ma (H.E.); kaoutar.abbassi@etu.uae.ac.ma (K.A.); maouni.safae88@gmail.com (S.M.); rahmouniqda@gmail.com (A.R.); mlegssyer@uae.ac.ma (M.L.); r.saidi@uae.ac.ma (R.S.); zh.amrani@yahoo.fr (Z.L.); amaouni@uae.ac.ma (A.M.); 2 Centro de Investigação em Química (CIQUP), Instituto de Ciências Moleculares (IMS), Departamento de Geociências, Ambiente e Ordenamento do Território, Faculdade de Ciências, Universidade do Porto, Rua do Campo Alegre s/n, 4169-007 Porto, Portugal; jcsilva@fc.up.pt; 3 Laboratory of Microbiology, Biological Sciences Department, Faculty of Pharmacy, University of Porto (FFUP), 4050-313 Porto, Portugal; 4 Interdisciplinary Centre of Marine and Environmental Research (CIIMAR), University of Porto, 4450-208 Matosinhos, Portugal

**Keywords:** *Tanacetum annuum*, chemical composition, total phenolic, total flavonoid, phenolic compounds, antioxidant activity, antifungal activity, *Fusarium oxysporum* f. sp. *albedinis*

## Abstract

*Tanacetum annuum* L. is a Mediterranean plant, commonly known as Blue Tansy due to its blue colour as an essential oil, which is widely used for medicinal purposes. However, there are no studies on the bioactive compounds (especially, phenolic compounds) and the biological properties of their organic extracts. Herein, the purpose of the present work was to investigate the chemical composition of the essential oil and crude extracts of the *T. annuum* aerial parts collected from northern Morocco and to evaluate their antioxidant and antifungal activity against *Fusarium oxysporum* f. sp. *albedinis*, an agent causing Bayoud disease of the date palm, an important food source and commercial perennial crop in the Sahara and North Africa. Chemically, Folin–Ciocalteu and aluminium chloride colourimetric methods were used to determine the total phenolic (TPC) and total flavonoid (TFC) contents, respectively; polyphenols were characterized using HPLC-MS, while GC-MS was used to analyse the essential oil composition. Moreover, the evaluation of antioxidant and antifungal activities was carried out using the DPPH test and microdilution method, respectively. The results showed that the three *T. annuum* parts (stems, leaves and flowers) extracts contained important TPC and TFC with values varied between 51.32 and 116.32 mg/g of dry crude extract (DCE). HPLC-MS analysis revealed the identification and quantification of 19 phenolic acids and flavonoids with an emphasis on apigenin 7-glucoside (4540 µg/g of dry weight (DW)), luteolin 7-glucoside (2804 µg/g DW) and salicylic acid (1878 µg/g DW). Additionally, 39 biomolecules were identified in the essential oil using GC-MS, which were predominated by camphor (16.69%), α-pinene (12.37%), bornyl acetate (11.97%) and limonene (11.10%). The methanolic and hydro-methanolic extracts of *T. annuum* parts demonstrated a strong antioxidant property with IC_50_ values ranging between 0.22 and 0.65 mg/mL. Concerning antifungal activity, the essential oil and crude extracts of the Moroccan Blue Tansy exhibited a potent capacity against *F. oxysporum* f. sp. *albedinis* at low concentrations, with MIC and MFC values of 3.33 and 4.58 µL/mL for the essential oil and values of 3.33 and 9.17 mg/mL for crude extracts, respectively. Overall, these results demonstrated *T. annuum* as an important source of bioactive compounds and contribute significantly to the potential of using essential oils and extracts for controlling the Bayoud disease of date palms. Moreover, the finding suggests that *T. annuum* can be highly useful for phytosanitary and pharmaceutical industries.

## 1. Introduction

The date palm (*Phoenix dactylifera* L.) is an important food source and commercial perennial crop in the Sahara and North Africa [1]. It plays essential economic and environmental roles in the oasis regions by creating a suitable microclimate for growing other food crops and protecting oases from silting and desertification [2]. In Morocco, the date palm has been cultivated on over 48,000 ha of traditional and genetically diverse oases and 20,000 ha of modern plantations cultivating commercial varieties in the southern part of the country [1,2]. One of the biggest destructive diseases of palm trees and a higher risk for the future of the date industrial sector in Morocco, as well as other countries producing dates, is commonly known in Morocco as “Bayoud”, which is a vascular disease, caused by a soil fungus, *Fusarium oxysporum* f. sp. *albedinis* (*Foa*), that blocks the circulation of raw sap in plants [2,3,4]. Bayoud was reported in Morocco for the first time in 1870, and it has been estimated that this fungus destroyed more than 10 million palm trees during the last 100 years [3,4]. Numerous strategies such as renewal of palm groves, genetic selection and fungicidal treatments have been applied to control Bayoud; however, they remain inefficient and difficult to apply [3,4]. Thus, there is an urgent requirement for discovering and developing new compounds that can control and combat Bayoud. Therefore, plants are an important natural source of secondary metabolites, such as alkaloids, terpenoids, phenolic compounds, glycosides, quinones and saponins, which play roles in the plant defence system against pathogen attacks during which their concentration in plants depends on pathosystem and environmental conditions [2].

Morocco is a country well-known for its richness and biodiversity of aromatic and medicinal plants, which are used in traditional medicine, by Moroccan populations, in a random way and with unidentified biomolecules and unknown concentrations. Among these plants, the *Tanacetum* species have been used for medicinal functions for decades due to their wide range of chemical, pharmacological and medicinal properties [5]. Accordingly, the species *Tanacetum annum* L. was chosen to conduct the present research.

*T. annuum* L. known as Blue Tansy, or as Moroccan blue chamomile, is an herbaceous plant that grows spontaneously in Mediterranean Bassan, and in particular in northwestern Morocco, Portugal, Spain and southern France. It belongs to the Compositae (or Asteraceae) family, which is considered the largest and within the most 20 prolific flowering plant families in the world [6]. *T. annuum* is used in traditional medicine as an anti-inflammatory, anti-histamine, anti-flogistic, and anti-pruriginic plant [7]; in addition, this plant has been tested for cytotoxic activity [7,8] and antifungal activity against a large spectrum of phytopathogenic fungi [9]. Nonetheless, to the best of our knowledge, and except for the essential oil, no research has been conducted on the chemical characterization of *T. annuum*, the antifungal activity against *Fusarium oxysporum* f. sp. *albedinis* (*Foa*), and the antioxidant activity. Herein, the objective of this study was first to investigate the chemical composition of essential oil and crude extracts of *T. annuum* collected from Northwestern Morocco, and secondly to evaluate the antioxidant (DPPH test) and antifungal activity (microdilution method) against *Fusarium oxysporum* f. sp. *albedinis*, an agent causing Bayoud disease of the Moroccan date palm, by using different extracts.

## 2. Results and Discussion

### 2.1. Extraction Yield

The extraction yield of essential oil (TaEO), crude methanolic (TaME) and hydro-methanolic (TaHME) extracts of *T. annuum* aerial parts are presented in Table 1. The obtained TaEO has a very dark blue color with a fruity and herbaceous aroma. Therein, the extraction yield of TaEO was on average 0.7% (wt/wt) of dry material, which was higher than the ones found previously with a percentage ranging between 0.23% and 0.5% [6,10,11], while our yield was noted to be lower than the studies by Greche et al. [12] and Zaim et al. [13] with the values of 0.8% and 1.2%, respectively.

Regarding the crude extracts, six extracts were prepared using three parts, including the stems, leaves and flowers, and two solvents, which were methanol (100%) and hydro-methanol (80:20, *v*/*v*). Generally, the results showed that hydro-methanol was the better solvent to obtain a high extraction yield in the three parts; in addition, the extraction yields of TaME and TaHME were closely similar to each other in the cases of flowers (26.66% and 28.90%) and leaves (20.07% and 21.89%), while in case of stems, the extraction yield of TaME was significantly lower than TaHME with values of 8.87% and 16.89%, respectively. These results demonstrated that extraction yields were affected by the plant part and solvent polarity used (Table 1). The polarity of solvents plays an important role in increasing extraction yield; whereas, the solvent with a higher polarity, such as water, has the ability of solubility and absorbance of bioactive compounds more than solvents with less polarity such as methanol, ethanol, etc. [14,15,16]. These confirmed the present results that showed a higher extraction yield using a combination of methanol with water than with methanol alone.

### 2.2. Bioactive Compounds Contents

The present work was the first study to estimate the bioactive compounds including total phenolic content (TPC), total flavonoid content (TFC) and carotenoid contents (lycopene (TLC) and β-carotene (Tβ-CC)) of the medicinal plant *T. annuum*. As presented in Table 2, the three parts of *T. annuum* contained important bioactive compounds, in particular, TPC and TFC in both crude extracts TaME and TaHME with values varying between 51.32 and 116.32 mg/g of dry crude extract (DCE). Similar to the extraction yield, the hydro-methanolic extracted higher bioactive compounds than the absolute methanol, with a significant difference between the values of the two solvent extracts in each part.

The TPC of *T. annuum* was observed to be significantly higher in the leaves than in the flowers and stems. The highest amount was found in the leaves TaHME and the lowest was in stems TaME with values of 116.32 and 51.32 mg of gallic acid equivalents (GAEs)/g of DCE, respectively. The TPC value in leaves TaME in the present work was higher than the one in *Tanacetum erzincanense* (64.4 mg GAEs/g DCE) previously reported by Yapıcı et al. [17]; in addition, the TPCs of the present study were higher than several *Tanacetum* species methanolic extracts, with their values varying from 32.15 to 47.11 mg GAEs/mg DCE [18]. However, Devrnja et al. [19] reported that methanolic extracts of *Tanacetum vulgare* from Serbia contained higher TPC than our finding in the three parts stems, leaves and flowers with the values of 83.60, 112.60 and 96.20 mg GAEs/g of DCE, respectively.

Concerning TFC, the highest content was observed in the leaves TaHME with the value of 91.54 mg of catechin equivalents (CEs)/g of DCE, and the lowest content was in the leaves and stems TaME with the amounts 58.81 and 58.63 mg/g of DCE, respectively. Flowers contained significantly higher TFC value in the case of TaME (64.04 mg CEs/g DCE) and the leaves in the case of TaHME (91.54 mg CEs/g DCE). In addition, there was no significant difference in the comparison between stems and leaves in the case of TaME and between stems and flowers in the case of TaHME. In comparison with the previous studies, our TFC values in *T. annuum* were observed to be higher than several other *Tanacetum* species that were reported to contain TFC values ranging from 18.54 to 55.40 mg/g of DCE, using different parts of the plants [18,20]. Yapıcı et al. reported that the TFC value of the methanolic extract of *T. erzincanense* was 62.20 mg of quercetin equivalents per g of DCE, which was higher than TaME and lower than TaHME of the leaves TFC of the current study [17].

Regarding the carotenoids (Table 2), lycopene was determined in the three parts of *T. annuum* with significantly different amounts from each other; whereas, stems contained the highest TLC, followed by leaves and flowers with the contents of 0.74, 0.69 and 0.19 µg/g of dry weight (DW), respectively. On the contrary, β-carotene was determined only in flowers with the value of 0.61 µg/g of DW.

Overall, the obtained results demonstrated that all parts of *T. annuum* contain an important amount of bioactive compounds, particularly total phenolic and total flavonoid, which possess a strong antioxidant activity and are responsible for many other biological activities. Moreover, the results demonstrated that bioactive compound contents were affected by solvent polarity and varied from one part to another. This verified what is described in the literature, the solvent with higher polarity extracted more bioactive compounds that are responsible for several bioactivities [14,15,16].

### 2.3. Chemical Composition of Essential Oil by GC-MS

**Figure 1:**
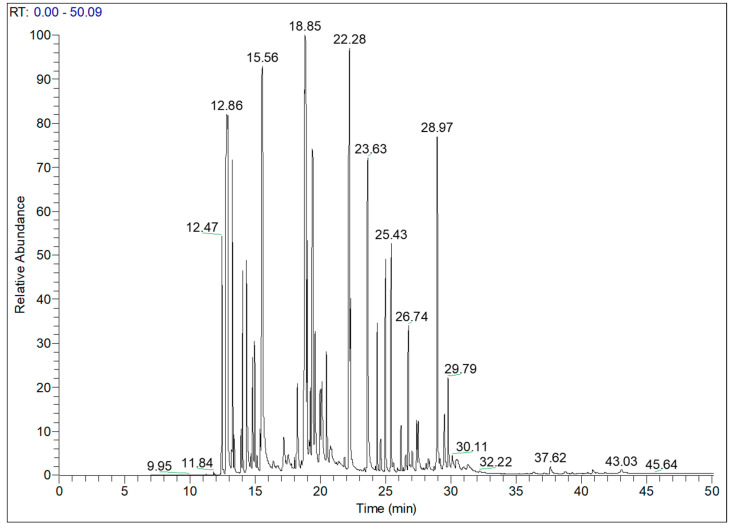
Chromatogram of *T. annuum* essential oil by GC–MS.

The chemical composition of TaEO of mixed aerial parts was analysed by GC-MS, and the results are illustrated in the chromatogram in Figure 1 containing 39 peaks which correspond to 39 identified compounds, representing 99.92% of total volatile essential oil compounds in *T. annuum*. These 39 biologically active molecules are divided into three classes of terpenoids: namely, monoterpenoids (85.22%), sesquiterpenoids (14.21%) and diterpenoids (0.49%). The main compounds identified were camphor (16.69%), α-pinene (12.37%), bornyl acetate (11.97%), limonene (11.10%), borneol (6.33%), α-terpinyl acetate (4.62%) and chamazulene (3.49%). The GC-MS results of TaEO are listed in Table 3.

The main compounds identified in *T. annuum* in the present study are already known for several biological activities. The predominant compound camphor, which possesses anti-inflammatory and antimicrobial activities is widely used in the cosmetic and pharmaceutical industries [6]; in addition, this compound showed strong activity against several phytopathogens of *Fusarium* species [21]. The second main substance, α-pinene, was noted to have antioxidant [22], anticancer, antimicrobial (including against *F. oxysporum* [23]) and anti-inflammatory activities [24]. Bornyl acetate has also been recorded to have antioxidant, antimicrobial, anti-inflammatory, anti-tumour and insecticidal properties [25]. Furthermore, the compound limonene is widely used in cosmetics, medicine and agriculture due to its activities such as antioxidant, anti-inflammatory, anticancer, insecticidal, antibacterial and herbicidal activity, and it is registered as a pesticide and a fungicide in China [26]. Moreover, borneol, α-terpinyl acetate and chamazulene were reported to possess antioxidant, anti-inflammatory and antifungal properties [6,24,25,27]. Additionally, in essential oils, even minor compounds can exert biological effects due to synergistic effects between chemical classes [25].

Several research works have investigated the chemical compositions of *T. annuum* essential oils using GC-MS analysis, and the majority of these works were from the northern region of Morocco, such as a study conducted by Greche et al., which indicated that sabinene (22.3%), camphor (13.2%) and β-pinene (10.1%) were the major components obtained from *T. annuum* aerial parts essential oil [9]. Zaim et al. reported that TaEO of aerial parts (leaves and flowers) collected from the same area of the study by Greche et al. [9] (Larache, northern Morocco) was dominated by the substances myrcene (13.67%), camphor (12.67%), sabinene (9.49%) and β-pinene (7.70%) [13]. In addition, chamazulene (17.74%), sabinene (14.39%) and camphor (14.21%) were the main compounds identified in TaEO by Belcadi et al. [6]. Moreover, a study using industrial TaEO from Germany found that the essential oil was rich in sabinene (14%), camphor (13.6%), myrcene (8%), β-pinene (7.7%), chamazulene (6.9%), α-phellandrene (6.5%) and *p*-cymene (5%) [5]. Similar to the GC-MS results of this study, all of the previous studies reported that camphor was one of the main compounds identified in TaEO. Overall, many factors can typically affect the quantity and chemical composition of the essential oil, including site and period of collection, stage of growth, and methods and conditions of drying, storage and extraction [28].

### 2.4. Characterization of Phenolic Compounds by HPLC-MS

**Figure 2:**
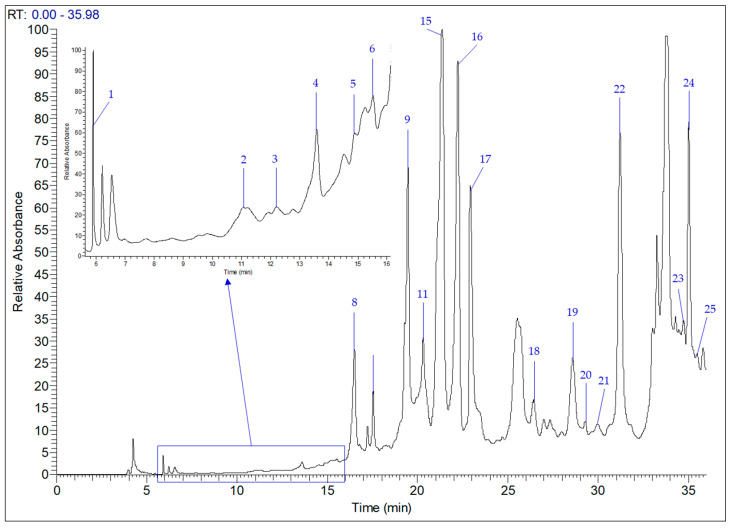
HPLC-MS chromatogram of individual phenolic compounds in the hydro-methanolic extract of *T. annuum* aerial part, as detected at 280 nm.

Phenolic compounds are natural bioactive compounds extracted mainly from plants that have demonstrated interesting biological activities, including antioxidant, anti-inflammatory, antimicrobial, and antiproliferative activities, among others, which has led to great interest in their use by numerous industries [29]. Therefore, the current study investigated, for the first time, the phenolic composition of the *T. annuum* plant using HPLC-MS. The individual phenolic compounds in the samples were identified and quantified based on their UV-Vis spectra, mass spectra, retention times and their areas by commercial standards [30]. Figure 2 demonstrates the HPLC-MS chromatogram illustrating the peaks of phenolic compounds present in the *T. annuum* aerial part, which were identified, quantified and presented in Table 4. From 19 identified compounds, 10 were phenolic acids (gallic, protocatechuic, chlorogenic, *p*-hydroxybenzoic, caffeic, syringic, ferulic acid, methylparaben, rosmarinic and salicylic acids) and 9 were flavonoids (catechin, rutin, luteolin 7-glucoside, apigenin 7-glucoside, luteolin, quercetin, apigenin and kaempferol). *T. annum* extract showed great content of individual polyphenols, which were mainly predominated by apigenin 7-glucoside (4540 µg/g DW), luteolin 7-glucoside (2804 µg/g), salicylic acid (1878 µg/g), rutin (786.40 µg/g), kaempferol (592.30 µg/g) and rosmarinic acid (579.9 µg/g), while gallic and caffeic acids were detected in the lowest amounts in the plant extract with the values of 11.29 and 7.46 µg/g, respectively. However, vanillic acid, ellagic acid, *p*-coumaric acid, vanillin, naringin and cinnamic acid were not detected in *T. annuum* hydro-methanolic extract.

The main compounds detected in the Blue Tansy (Moroccan Blue Chamomile) are well-known for several biological effects; for instance, the major flavonoid identified, apigenin 7-glucoside, a widely distributed food flavone in the genus of *Tanacetum,* has shown numerous biological properties, including antioxidants, anti-inflammatory, antiviral, antifungal and hepatoprotective [31,32]. The following flavonoid luteolin 7-glucoside, a primary active component of luteolin, has demonstrated multiple biological activities such as antimicrobial, antioxidant, anti-inflammatory and antitumor [33,34]. In addition, the third main identified compound salicylic acid is a phenolic acid well-known for its medicinal impact on a variety of skin disorders and contributes to skin exfoliation and removal of dead cells [35,36].

As there is no literature on phenolic compounds of *T. annuum*, the HPLC-MS results of this work will be compared with *T. vulgare,* which is a species closer to ours, and also compared with other *Tanacetum* such as *T. balsamita* and *T. parthenium*. Herein, the studied plant was more rich and diversified in polyphenols than the *Tanacetum* species cited above [20,34,37]. Bączek et al. found that *T. vulgare* and *T. balsamita* contained a higher concentration of phenolic acids than flavonoids which is contrary to our findings; however, apigenin-7-O-glucoside and luteolin 7-O-glucoside was observed as the main flavonoids in *T. balsamita,* which agrees with this study [37]. In the study by Babich et al., chlorogenic acid, ferulic acid and rosmarinic acid were the main phenolic compounds identified in *T. vulgare* from Russia [34]. The leaves of *Tanacetum* species *T. vulgare*, *T. macrophyllum*, and *T. corymbosum* as well as the aerial parts of *T. parthenium* have recently been found to have high levels of chlorogenic acid [20,38]. Generally, the diversity and richness of phenolic compounds differ from one plant to another. Overall, the aerial part of the investigated plant *T. annuum* can be considered a great natural source of polyphenols, which are important for the development of several agricultural and pharmaceutical products.

### 2.5. Antifungal Activity and Synergistic Effect

The antifungal activity of *T. annuum* crude extracts and essential oil was carried out against the fungal pathogen *Fusarium oxysporum* f. sp. *albedinis* (*Foa*) that causes Bayoud disease, vascular wilt in date palm, which is considered a major constraint that impedes the development of the date palm sector in North Africa and Sahara, and more specifically in Morocco [39]. This research revealed that all *T. annuum* extracts tested effectively inhibited the growth of *Foa*, indicating the presence of strong antifungal compounds. The minimum inhibitory concentration (MIC) and minimum fungicidal concentration (MFC) values of different tested samples are listed in Table 5; whereas the concentrations required to cause growth inhibition of *Foa* ranged from 3.33 to 9.17 mg/mL by crude extracts, and from 0.5 to 4 mg/mL by commercial fungicides and its combination with essential oil. Moreover, the best growth inhibition result was obtained by essential oil alone with MIC and MFC values of 3.33 and 4.58 µL/mL, respectively. Concerning the crude extracts, the leaves showed the highest anti-*Foa* property with an MIC of 3.33 mg/mL for both solvents and an MFC of 7.50 and 8.33 mg/mL for TaHME and TaME, respectively. The anti-*Foa* activity of extracts from the stems and flowers was slightly similar. In addition, generally speaking, there were no significant differences between TaME and TaHME of each part, these could be due to their similarity and no higher difference in the amounts of bioactive compounds such as total phenolic and total flavonoids or individual polyphenols, which are already known for their antimicrobial activity [29]. Therefore, as there was a highly similar amount of total phenolic and total flavonoids between the three parts and by using the two solvents, the major individual phenolic compounds (namely, apigenin 7-glucoside, luteolin 7-glucoside, salicylic acid, rutin, kaempferol, rosmarinic acid, ferulic acid, quercetin, syringic acid and catechin) found in the mixed aerial parts of *T. annuum* could be the main phytochemical contributors for the growth inhibition of the harmful pathogen *Foa* by acting alone or in synergy. In terms of mechanisms of action, phenolic acids and flavonoids can exhibit antifungal activity by disrupting cell division, hyphal formation and/or triggering severe oxidative stress leading to cell death. This may be attributed to the direct impact of active phytochemical ingredients on fungal cells or due to the induction of defense mechanisms in the host plant [40,41].

Regarding commercial fungicides which are used against Bayoud diseases, the results demonstrated that SAAF 75% inhibited the growth of *Foa* better than carbendazim 50% with a half dose. As presented in Table 6, the results obtained on the synergistic effects of TaEO in combination with the two used synthetic fungicides showed a synergistic interaction in the association with carbendazim 50% with an FICI value of 0.4, while showing an additive interaction in the case of the combination between TaEO and SAAF 75% with an FICI value of 0.65. The combination of TaEO (3 µL/mL) with the used fungicides (1 mg/mL) reduced the dose for inhibition of the pathogen in half for SAAF 75% and by two thirds for carbendazim 50% (Table 5). The combination of individual compounds, essential oil and other antifungal agents possess particular benefits for antimicrobial activity, and their mechanism of action has multiple targets on microorganisms such as cell wall destruction, increasing permeability, damaging of the cytoplasmic membrane, membrane protein damage, and coagulation of cytoplasm resulting in metabolic damage and cell death [42]. Generally, this combination could be an interesting alternative to combat Bayoud diseases.

The strong antifungal activity of *T. annuum* essential oil against *Foa* could be attributed to its major compounds such as camphor which possessed several biological activities [6], in particular, a strong activity against several phytopathogens of the *Fusarium* species [21] and the major substance α-pinene noted to have activity against *F. oxysporum* [23]. Additionally, bornyl acetate and limonene have been recorded as having antimicrobial and insecticidal properties, and limonene is used as a fungicide [25,26]. In addition to that, the other main compounds, borneol, α-terpinyl acetate and chamazulene, have been reported to have the ability of growth inhibition of fungal pathogens [6,24,25,27], and not forgetting that in essential oil, even the minor compounds can exert biological effects due to synergistic effects between chemical classes [25]. Concerning the mechanism of action of essential oil, the antifungal activity of TaEO might be caused by the properties of terpenes/terpenoids that—due to their highly lipophilic nature and low molecular weight—are capable of disrupting the cell membrane, causing cell death or inhibiting the sporulation and germination of food spoilage fungi [43,44], which also can be caused due to synergistic effects between chemical classes [25]. Furthermore, several studies on antifungal activity reported that *T. annuum* essential oil has been able to inhibit the growth of numerous fungal species at different concentrations, including *Alternaria solani*, *Botrytis cinerea*, *Helminthosporium oryzae*, *Pyricularia oryzae*, *Verticillium dahliae* [9], *Penicillium expansum* [6], *Aspergillus niger*, *Candida albicans* and *Cryptococcus neoformans* [45].

Several research works investigated the antifungal activity of numerous plants’ crude extracts and essential oils against *F. oxysporum* f. sp. *albedinis* [1,2,3,46,47]. The study by Rahmouni et al. investigated the antifungal activity of essential oils of five medicinal and aromatic plants collected from northern Morocco against the same pathogen isolate *F. oxysporum* f. sp. *Albedinis,* and the results demonstrated that *Origanum compactum* (MIC = 2.5 and MFC = 5 µL/mL) showed better activity than the four plants *Myrtus communis*, *Thymus satureioides*, *Lavandula dentata* and *Rosmarinus officinalis* with MIC and MFC ranges between 10 and 40 µL/mL; in addition, these last four plants were also less active against *Foa* than the present essential oil [3]. On the other hand, Rahmouni et al. also evaluated the activity of the major components of its plant which included three molecules present in the current analysed essential oil as major compounds, namely, α-pinene, borneol and with a small percentage of α-terpineol, and these compounds possessed an anti-*Fusarium* effect [3]. The previous studies by Chibane et al. [47] and Chibane et al. [1] cited, respectively, that the essential oil of *Cladanthus eriolepis* and *Asteriscus graveolens* showed growth inhibition values of 86.20% and 100% against *Foa* at the concentration of 4 µL/mL, which is slightly higher than our concentration. Concerning the crude extracts, Bouhlali et al., previously determined the antifungal activity of five plant extracts, namely, *Acacia cyanophylla*, *Cupressus atlantica*, *Eucalyptus torquata*, *Nerium oleander* and *Schinus molle,* against *Foa*, and results showed that all extracts possessed antifungal activity, where the extracts of *E. torquata* and *C. atlantica* showed the strongest antifungal effect resulting in the inhibition of mycelial growth, sporulation and spore germination in a dose-dependent manner [2].

Overall, the crude extracts and essential oil of *T. annuum* showed a strong antifungal activity due to the ability of their major compounds to inhibit the growth of the pathogen. These compounds will be our future work to separately evaluate their anti-*Fusarium* activity and to understand the mechanisms and their connection with their antifungal action.

### 2.6. Antioxidant Activity

**Figure 3:**
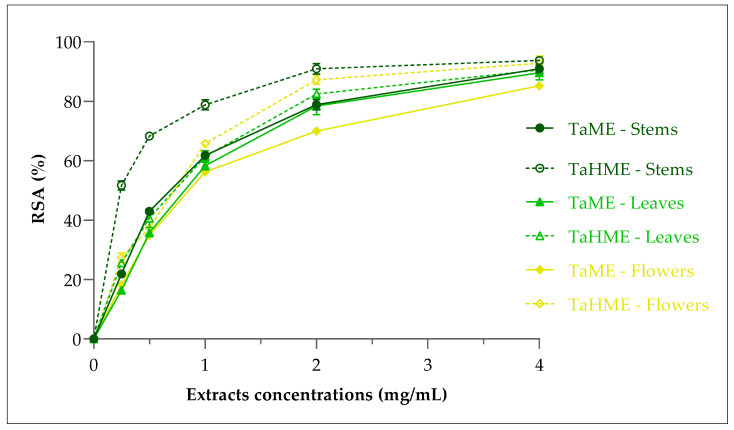
Radical-scavenging activity (RSA) on DPPH radicals of *T. annuum* crude extracts. Values are expressed as the mean ± SD (*n* = 3).

The antioxidant activity of the crude extracts TaME and TaHME of the *T. annuum* parts (stems, leaves and flowers) was evaluated using the DPPH (2,2-Diphenyl-1-picrylhydrazyl) assay, which is valid, accurate, quick, easy, economic and one of the most widely used methods for assessing the antioxidant properties of the extracts or purified compounds [48]. Figure 3 illustrates the increase of DPPH radical activities of the six extracts with the increase of the tested samples’ concentration (ranging from 0.25 to 4 mg/mL). Based on radical-scavenging activity inhibition (%), IC_50_ values of the samples were graphically determined by their concentration providing 50% inhibition values (Figure 3 and Table 7). Each high IC_50_ value indicates a lower antiradical potential [49].

As shown in Table 7, all the crude extracts exhibited a great antioxidant capacity as expected, considering the high amounts of total phenolic and total flavonoid contents in each part extract. The IC_50_ values ranged between 0.22 and 0.65 mg/mL. The highest antioxidant effect was shown by TaHME (stems) with a value of 0.22 mg/mL, and the lowest one was observed in TaME (leaves) with a concentration of 0.65 mg/mL; in addition, there was no significant difference between the leaves’ and flowers’ antioxidant capacity in the two extract cases, TaME and TaHME. However, the antioxidant activity of the TaHME extracts was slightly better than TaME in the three parts (Table 7). Ascorbic acid was used as a positive control and showed stronger antioxidant properties with the IC_50_ value equal to 0.036 mg/mL. The phenolic compounds including phenolic acids and flavonoids are considered primary antioxidants, which have the ability to neutralize the free radicals or inhibit the production of free radicals from hydroperoxides by donating hydrogen atoms to lipid radicals [50]. The mechanism of antioxidant actions involved either hydrogen atom transfer, transfer of a single electron, sequential proton loss electron transfer, or chelation of transition metals [51].

To our knowledge, the antioxidant potential of different parts of *T. annuum* has not been evaluated by any previous studies. For this reason, comparisons will be made with other *Tanacetum* species. Herin, several studies investigated the antiradical activity of *Tanacetum* species using aerial parts mixed or separated and different solvents; therefore, their results varied from one sample to another. Baranauskienė et al. performed the antioxidant activity of *T. vulgare,* which gave the best result using water extract with EC_50_ = 1.33 mg/mL (EC = effect concentration); this finding was lower than the one obtained in the current study [52]. *T. vulgare* was also the objective of a study by Devrnja et al., investigating the antioxidant activity of methanolic extracts of stems, leaves and flowers, with obtained IC_50_ values of 0.080, 0.077 and 0.058 mg/mL, respectively [19]. Furthermore, Esmaeili et al. [18] evaluated the antioxidant properties of the ethanolic extracts of the aerial part of six *Tanacetum* species (*T. budjnurdense*, *T. hololeucum*, *T. chiliophyllum*, *T. sonboli*, *T. tabrisianum* and *T. kotschyi*), and the results showed IC_50_ values ranged between 0.060 and 0.157 mg/mL, which were better than our findings. Overall, the Moroccan *T. annuum* could be considered a good source of antioxidant compounds.

## 3. Materials and Methods

### 3.1. Chemical Material

Alkane standards (C_8_–C_20_ and C_21_–C_40_), Folin–Ciocalteu phenol reagent, l-ascorbic acid, dimethyl sulfoxide (DMSO), PDA (Potato Dextrose Agar), RPMI-1640 and all individual phenolic compounds were obtained from Sigma-Aldrich, Co., (St. Louis, MO, USA). Acetonitrile, diethyl ether and ethyl acetate were purchased from Merck KGaA (Darmstadt, Germany). Acetone, hexane and n-hexane were acquired from CABLO ERBA Reagent, S.A.S (Val de Reuil Cedex, France). SAAF (Mancozeb 63% + Carbendazim 12%) was obtained from LAKORALE (Casablanca, Morocco) and GOLDAZIM 500 SC = 500 g/L (Carbendazim 50%) from PHILEA (Casablanca, Morocco). DPPH (2,2-diphenyl-l-picrylhydrazyl) was obtained from Alfa Aesar (Ward Hill, MA, USA). Methanol and all other solvents were purchased from Honeywell (St. Muskegon, MI, USA).

### 3.2. Plant Material

The aerial parts (stems, leaves and flowers) of the plant *Tanacetum annuum* were harvested during the flowering period of July–September 2020 at the Biological and Ecological Interest Site (SIBE) of Ben Karrich, a site of biological and ecological interest belonging to the Tetouan province in northwestern Morocco (35°30′50.6″ N. 5°26′13.9″ W, 220 m of altitude, thermo-Mediterranean vegetation level, a subhumid bioclimatic level at temperate winter, and siliceous substrate). The plant identification was based on the name given by specialists in mass cultivation of this plant and confirmed in our laboratory using the determination key “Practical Flora of Morocco: Manual of Determination of Vascular Plants” [53]. The different aerial parts of the plant were dried in the shade at room temperature for 7 days. They were then crushed, and the resulting fine powder (20 mesh) was stored in a dark glass bottle until the preparation of the plant’s EO and crude extracts.

### 3.3. Preparation of Essential Oil and Crude Extracts

The isolation of the TaEO was made from the aerial parts of the *T. annuum* by hydrodistillation for 3 h, using a Clevenger-type apparatus, where 100 g of fine powder was immersed in a 1000 mL flask of distilled water. The obtained oil was stored at −25 °C in a dark glass vial until use.

The crude extract preparation of the three parts, stems, leaves and flowers, was carried out by using two solvents, methanol and hydro-methanol (methanol/water, 80:20, *v*/*v*), and following a procedure used previously by Erbiai et al. [54] with some modifications. One gram of powder of each part was added to 10 mL of used solvents; then, the mixture was agitated using a magnetic stirrer at room temperature for 24 h and filtered through Whatman grade 4 qualitative filter paper. The unfiltered part was again extracted two times using the same procedures. The combined extracts were evaporated under reduced pressure at 40 °C by using a rotary vacuum evaporator to give dry extracts, which were then weighed and stored at −25 °C for further use.

The preparation of TaEO and *T. annuum* crude extracts was performed in triplicate and the extraction yield was calculated for each sample.

### 3.4. GC-MS Analysis of Essential Oil

The chemical components of TaEO were characterized by GC-MS, following the protocol described early by Erbiai et al. [55]. Briefly, a volume of 10 µL of TaEO was diluted in 90 µL of n-hexane and analysed using gas chromatography (GC) (Trace 1300 gas chromatograph; Thermo Fisher Scientific, Waltham, MA, USA) coupled with mass spectrometry (MS) (ISQ single quadrupole mass spectrometer; Thermo Fisher Scientific). The GC was equipped with the TG5-MS capillary column (60 m × 0.25 mm i.d.; 0.25 µm film thickness) with a non-polar stationary phase (5% phenyl and 95% dimethylpolysiloxane). The oven temperature was programmed to increase from 40 °C to 350 °C at a rate of 5 °C/min. The carrier gas used was helium with a flow rate of 1.2 mL/min. The components of TaEO were identified based on Kovats retention indices (RI) relative to those of a homologous series of known standards of alkanes mixture (C_8_–C_20_ and C_21_–C_40_) and with the spectral data obtained from the National Institute Standard and Technology (NIST) databases of the corresponding substance. Data acquisition and analysis were performed by Software Thermo XcaliburTM 2.2 SP1.48 and NIST MS Search 2.2 Library 2014, respectively.

### 3.5. Determination of Bioactive Compound Contents

The content determination of total phenolic (TPC), total flavonoid (TFC) and total carotenoids (β-carotene (Tβ-CC) and lycopene (TLC)) in *T. annuum* leaves and stems crude extracts was carried out using spectrophotometric methods [54].

TPC determination was performed by following the Folin–Ciocalteu method. A total of 1 mL of each crude extract solution (1 mg/mL) was mixed in a tube with 5 mL of Folin–Ciocalteu reagent (diluted at 1/10) and 4 mL of sodium carbonate (7.5%). The tube was mixed by vortex for 15 s and allowed to react by standing in the dark for 30 min at 40 °C. Afterwards, the mixture absorbance was measured at 760 nm against a blank. Gallic acid was used as a standard for obtaining the calibration curve and the results of different samples were expressed in mg of gallic acid equivalents (GAE) per gram of dry crude extract (DCE).

TFC was estimated by the aluminium chloride colourimetric method. Herein, a volume of 250 µL of each diluted plant extract (1 mg/mL) was mixed with 1100 µL of distilled water and 75 µL of NaNO_2_ solution (5%). After 5 min, 75 µL of AlCl_3_ solution (10%) was added and allowed to stand for a further 6 min; then, 1000 µL NaOH solution (4%) was added and mixed by vortex apparatus. The mixture was allowed to stand for another 15 min before measuring the absorbance against the blank at 510 nm. The TFC existing in the extracts was qualified using the standard calibration curve of flavonoid, (+)-catechin, and the results were expressed as mg of catechin equivalents (CE)/g of DCE.

The concentration of β-carotene and lycopene for each extract was determined following a procedure previously described by the authors Nagata and Yamashita [56]. Two hundred grams of fine powder was mixed with 5 mL of acetone–hexane mixture (4:6) for one minute. The solution was filtrated through Whatman N° 4 paper and the absorbance (A) was measured at 453, 505, 645, and 663 nm. The amounts of β-carotene and lycopene were determined using the following the equations: β-carotene (mg/100 mL) = [(0.216 × A_663_) − (1.22 × A_645_) − (0.304 × A_505_) + (0.452 × A_453_)] and Lycopene (mg/100 mL) = [(−0.0458 × A_663_) + (0.204 × A_645_) + (0.372 × A_505_) − (0.0806 × A_453_)].

### 3.6. HPLC-MS Analysis of Individual Phenolic Compounds

#### 3.6.1. Phenolic Compounds Extraction

The extraction of phenolic compounds was carried out by following a procedure described by the authors Erbiai et al. [57] with some modifications, depending on our available materials. One gram of fine powder of *T. annuum* aerial parts was extracted with 20 mL of hydro-methanol (methanol/water, 80:20, *v*/*v*) at −20 °C for 2 h. Subsequently, the mixture was shaken for one hour using a magnetic stirrer in the dark and then centrifuged at 3000 rpm for 10 min. The mixture was filtered through Whatman N° 4 paper and the residue was re-extracted once more under the same conditions. The combined extracts were evaporated under reduced pressure at 40 °C to remove the methanol. Afterwards, 20 mL of diethyl ether and 20 mL of ethyl acetate were used twice to submit the aqueous phase via liquid–liquid extraction. After adding anhydrous sodium sulfate to the combined organic phase, the extract was filtered through Whatman N° 4 paper and evaporated at 40 °C to dryness. A total of 5 mg of the obtained extract was dissolved in 1 mL of methanol: water (80:20, *v*/*v*) and passed through a 0.22 µm disposable LC filter disc for HPLC analysis.

#### 3.6.2. HPLC-MS Analysis

The identification and quantification of individual phenolic compounds existing in the aerial parts of *T. annuum* were performed according to the method previously described by the authors Erbiai et al. [57], using similar settings and HPLC equipment. Briefly, the polyphenolic extract was characterized by high-performance liquid chromatograph–mass spectrometry (HPLC-MS). The chromatographic separation was carried out using an Acclaim™ 120 reverse phase C18 column (3 µm 150 × 4.6 mm) thermostatted at 35 °C, with 280 nm as the preferred wavelength for peak detection of phenolic compounds. The mobile phase consisted of 1% acetic acid (A) and 100% acetonitrile (B). The individual phenolic compounds in the sample were identified based on their UV–Vi’s spectra and by comparing their mass spectra and retention times with those of commercial standards. The recorded peaks at 280 nm were quantified by calculating their areas and comparing them to the calibration curve obtained from the standard of each compound [30]. The final results were expressed in µg per gram of dry weight (DW).

### 3.7. Evaluation of Antifungal Activity

#### 3.7.1. Fungal Pathogen and Growth Conditions

The fungal pathogen responsible for Bayoud disease, *Fusarium oxysporum* f. sp. *albedinis* (*Foa*), was isolated from the soil around the root and leaves of a date palm tree *Phoenix dactylifera* L. with the symptoms in a Moroccan Oasis located in the Errachidia region. The collection, isolation and identification of the *Foa* were performed by the authors Rahmouni et al. [3] in our laboratory. The fungus isolate was cultivated in vitro on Potato Dextrose Agar (PDA) medium for 5–8 days at 25 °C, and the fungal culture was routinely subjected to a series of successive purifications until a pure mycelial was ready to use; then it was conserved at −86 °C. The identification of *Foa* isolate was made based on the morphological and cultural characteristics. This was mainly accomplished through the microscopic observation of macroconidia shapes and sizes and the mycelial structure as well as by using pigmentation and growth rates on agar media in comparison with previous studies [3,58]. Moreover, the obtained pure culture was tested on cultures of tomato and date palm to confirm pathogenicity and identification of the *Foa* isolate and its involvement in the Bayoud outbreak.

#### 3.7.2. Determination of MIC and MFC Values and Synergistic Effects

The antifungal activity of *T. annuum* essential oil and different crude extracts against *Foa* was performed by determination of MIC and MFC values, using a microdilution technique recommended by the Clinical and Laboratory Standard Institute-CLSI 2008 (M38-A2, filamentous fungi) and previously described in published works by our team [3,57,59]. Briefly, a stock solution of each crude extract (TaMEs and TaHMEs), TaEO, synthetic fungicides (SAAF 75% (mancozeb 63% + carbendazim 12%) and carbendazim 50%) and a combination of TaEO with synthetic fungicides, were prepared in dimethyl sulfoxide (DMSO) and diluted in sterile RPMI 1640 at 5% of the final solution of DMSO to reach the appropriate concentrations to be assayed. A volume of 60 µL of each prepared concentration was poured into the well of a U-bottomed 96-well plate together with 60 µL of *Foa* final spore suspension of approximately 10^4^ spores/mL prepared in RPMI. The final tested concentrations for TaEO, crude extracts and synthetic fungicides were 10.00–0.09 µL/mL, 20.00–0.63 mg/mL and 4.00–0.06 mg/mL, respectively. The synergistic test of TaEO combined with synthetic fungicides was evaluated by adding 3 µL/mL of TaEO to the stock solution of fungicides (1 mg/mL), and then a serial dilution was achieved to reach the final tested concentration ranging between 1 and 0.06 mg/mL. Controls consisted of sterility, with RPMI-1640, and growth, with spores’ suspension and RPMI-1640 medium plus DMSO (5%). Thereafter, the 96-well microplates were incubated for 48 h at 25 °C and the lowest concentration that totally inhibited the spore germination of *Foa* was considered the MIC value. The determination of MFC values was accomplished by inoculation of 10 µL from those wells showing no turbidity in determination of MIC, into a Petri dish on PDA medium. The MFC value was determined as the lowest concentration that totally inhibited the mycelial growth of *Foa* under incubation for 7 days at 25 °C.

The fractional inhibitory concentration index (FICI) of the combination of TaEO with synthetic fungicides (SF) was calculated based on the sum of the fractional inhibitory concentration (FIC) as shown in the following equation [60]:

$$FIC\;of\;TaEO=\frac{MIC\;of\;TaEO\;in\;Combination}{MIC\;of\;TaEO\;Alone}$$

$$FIC\;of\;SF=\frac{MIC\;of\;SF\;in\;Combination}{MIC\;of\;SF\;Alone}$$

FICI=FIC of TaEO+FIC of SF

FICI values were interpreted according to Afeltra et al. [61]: FICI ≤ 0.5 ‘synergistic’; 0.5 < FICI ≤ 1.0 ‘additive’; 1.0 < FICI ≤ 4.0 ‘indifferent’; and FICI > 4.0 ‘antagonistic’.

### 3.8. Evaluation of Antioxidant Activity

The antioxidant activity of Blue Tansy crude extracts was evaluated using an assay of free DPPH^•^ (2,2-diphenyl-1-picrylhydrazyl) radical-scavenging activity (RSA) using a modified version of the methodology outlined in previous studies [54,62]. A volume of 300 µL of the plant samples or standard (ascorbic acid) at concentrations ranging from 4 to 0.25 and 1 to 0.06 mg/mL, respectively, was added to 2.7 mL of methanol solution of DPPH. The mixture was incubated in darkness for 30 min at room temperature, subsequently, the absorbance was measured at 517 nm using a UV-Vis spectrophotometer against blank. The percentage of DPPH scavenging activity was calculated as % inhibition of DPPH using the following equation: RSA (%) = [(A*_DPPH_* − A*_Sample_*)/A*_DPPH_*] × 100. Samples or standard concentration providing 50% DPPH scavenging (IC_50_) was graphically calculated. Ascorbic acid was used as a reference standard in this study.

### 3.9. Data Analysis

All the measurements were made in triplicate, and the results were expressed as mean ± standard deviation (SD). The statistical significance was calculated by the application of a one-way ANOVA test, followed by post hoc Tukey’s multiple comparison tests with *p* ≤ 0.05 level considered significant, by using GraphPad Prism 8.0.1 software.

## 4. Conclusions

Bayoud, the biggest destructive disease of palm trees by the fungal pathogen *Fusarium oxysporum* f. sp. *albedinis* (*Foa*) urgently requires the discovery and development of new compounds that can control or aid in controlling this disease. The present study permitted evaluation for the first time of the crude extracts’ chemical composition of *Tanacetum annuum* aerial parts; proved the antioxidant activity of extracts; and demonstrated the antifungal activity of extracts and essential oil against Bayoud disease (*Foa*). The results proved that the plant extracts contained important bioactive compounds, mainly the phenolic compounds (total and individual (19 substances)), as well as the essential oil being composed of 39 volatile compounds classed mostly into the monoterpenoids class (85.22%). This richness and variety of compounds present in *T. annuum* has contributed strongly to antioxidant and anti*-Foa* activity. The crude extracts demonstrated a strong antioxidant property with small IC_50_ values. Regarding anti-*Foa*, all extracts of *T. annuum* exhibited potent capacity at low concentrations. Therefore, it can be suggested that *T. annuum* essential oil, crude extracts and a combination of essential oil and commercial fungicides, which showed a strong antifungal capacity, could be a useful alternative to protect and combat fungal diseases, in particular *Foa*. However, further study is required to evaluate the anti-*Foa* activity of the major compounds identified in *T. annuum,* separately and/or in combination, and to understand the mechanisms and their connection with their antifungal action.

## Figures and Tables

**Table 1:** Extraction yield (%) of TaEO and crude extracts of *T. annuum*.

Extracts	*T. annuum* Parts		
	Stems	Leaves	Flowers
TaEO	0.7 ± 0.01 *		
TaME	8.87 ± 0.19 ^Bc^	20.07 ± 0.30 ^Bb^	26.66 ± 0.35 ^Aa^
TaHME	16.89 ± 0.16 ^Ac^	21.89 ± 0.19 ^Ab^	28.90 ± 0.18 ^Aa^

* Essential oil of the mixed aerial parts. Values are the mean ± standard deviation (SD) for three replicates. For each part and extract, different superscript uppercase and lowercase letters indicate statistically different means within each column and row (*p* < 0.05), respectively.

**Table 2:** Bioactive compounds of *T. annuum* flowers, leaves and stems.

Bioactive Compounds		*T. annuum* Parts		
		Stems	Leaves	Flowers
TPC (mg GAEs/g DCE)	TaME	51.32 ± 0.82 ^Bc^	87.81 ± 0.41 ^Ba^	63.81 ± 0.39 ^Bb^
	TaHME	97.58 ± 0.41 ^Ab^	116.32 ± 0.60 ^Aa^	85.52 ± 0.35 ^Ac^
TFC (mg CEs/g DCE)	TaME	58.63 ± 0.00 ^Bb^	58.81 ± 0.70 ^Bb^	64.04 ± 0.36 ^Ba^
	TaHME	77.24 ± 0.35 ^Ab^	91.54 ± 0.36 ^Aa^	76.89 ± 0.73 ^Ab^
Carotenoids (µg/g DW)	TLC	0.74 ± 0.00 ^a^	0.69 ± 0.01 ^a^	0.19 ± 0.00 ^b^
	Tβ-CC	0.00	0.00	0.61 ± 0.00

Values are the mean ± SD for three replicates. For each part and extract, different superscript uppercase and lowercase letters indicate statistically different means within each column and row (*p* < 0.05), respectively.

**Table 3:** The chemical constituents of the essential oil of *T. annuum* obtained by GC-MS analysis.

N°	Compound Name	RI *	Area (%)
1	Tricyclene	925	2.53
2	α-Pinene	937.66	12.37
3	Camphene	951.3	2.77
4	Dehydrosabinene	955.52	0.39
5	Sabinene	972.73	0.38
6	β-Pinene	976.95	1.54
7	β-Myrcene	986.04	2.61
8	α-Phellandrene	1001.89	1.2
9	3-Carene	1007.58	1.1
10	o-Cymene	1024.24	0.38
11	Limonene	1029.55	11.1
12	m-Menthane	1092.42	0.69
13	α-Campholenal	1132.81	1.39
14	Camphor	1156.92	16.69
15	Pinocarvone	1171.94	0.85
16	Borneol	1178.66	6.33
17	Terpinen-4-ol	1186.56	2.02
18	α-Terpineol	1201.67	1.17
19	Myrtenal	1207.53	1.33
20	Verbenone	1222.18	1.26
21	cis-p-Mentha-1(7),8-dien-2-ol	1234.31	0.45
22	Isopulegol acetate	1280.33	0.08
23	Bornyl acetate	1296.65	11.97
24	α-Terpinyl acetate	1357.59	4.62
25	α-Copaene	1390.18	1.09
26	(-)-β-Bourbonene	1401.88	0.35
27	α-Gurjunene	1419.25	1.7
28	Caryophyllene	1439.91	1.81
29	Humulene	1475.12	0.42
30	γ-Muurolene	1492.49	0.66
31	Germacrene D	1501.51	1.35
32	α-Amorphene	1516.58	0.28
33	δ-Cadinene	1539.7	0.59
34	Caryophyllene oxide	1637.91	0.86
35	Chamazulene	1712.78	3.49
36	Cubenol	1751.66	1.08
37	Aromadendrene oxide 2	1785.08	0.53
38	Manoyl oxide	2028.1	0.2
39	Totarol	2346.58	0.29
1–24	Monoterpenoids		85.22
25–37	Sesquiterpenoids		14.21
38–39	Diterpenoids		0.49
	Total		99.92

* RI: Kovats retention indices.

**Table 4:** Phenolic compounds identified and quantified in the hydro-methanolic extract of the *T. annuum* aerial part.

Peak N°.	Phenolic Compounds	Contents (µg/g DW)
1	Gallic acid	11.29 ± 0.10
2	Protocatechuic acid	26.39 ± 0.29
3	Chlorogenic acid	79.95 ± 1.42
4	Catechin	298.9 ± 1.16
5	*p*-Hydroxybenzoic acid	24.37 ± 0.77
6	Caffeic acid	7.46 ± 0.10
7	Vanillic acid	ND
8	Syringic acid	300.3 ± 1.34
9	Rutin	786.4 ± 7.38
10	Ellagic acid	ND
11	Luteolin 7-glucoside	2804 ± 13.11
12	*p*-Coumaric acid	ND
13	Vanillin	ND
14	Ferulic acid	427.2 ± 2.36
15	Naringin	ND
16	Apigenin 7-glucoside	4540 ± 4.45
17	Rosmarinic acid	579.9 ± 5.18
18	Salicylic acid	1878 ± 1.23
19	Methyl paraben	263.2 ± 0.52
20	Luteolin	143.8 ± 0.93
21	Quercetin	321.5 ± 2.82
22	Cinnamic acid	ND
23	Apigenin	114.1 ± 3.56
24	Kaempferol	592.3 ± 3.69
25	Isorhamnetin	154.8 ± 3.28

Values are the mean ± SD for three replicates. ND—not detected.

**Table 5:** MIC and MFC values of the antifungal activity of *T. annuum* essential oil and crude extracts and reference fungicides against date palm Bayoud disease by *F. oxysporum* f. sp. *albedinis* (*Foa*).

Samples		MIC (mg/mL)	MFC (mg/mL)
Stems	TaME	5.83 ± 2.04 ^a^	9.17 ± 2.04 ^a^
	TaHME	5.83 ± 2.04 ^a^	9.17 ± 2.04 ^a^
Leaves	TaME	3.33 ± 1.29 ^b^	8.33 ± 2.58 ^ab^
	TaHME	3.33 ± 1.29 ^b^	7.50 ± 2.74 ^b^
Flowers	TaME	5.00 ± 0.00 ^a^	9.17 ± 2.04 ^a^
	TaHME	5.00 ± 0.00 ^a^	8.33 ± 2.58 ^ab^
Aerial part	TaEO (µL/mL)	3.33 ± 1.29	4.58 ± 1.02
Synthetic fungicides	SAAF 75%	1.00 ± 0.00 ^cd^	2.00 ± 0.00 ^d^
	Carbendazim 50%	2.00 ± 0.00 ^c^	4.00 ± 0.00 ^c^
Synthetic fungicides+ TaEO (3 µL/mL)	SAAF 75% + TaEO	0.50 ± 0.00 ^d^	1.00 ± 0.00 ^e^
	Carbendazim 50% + TaEO	0.50 ± 0.00 ^d^	1.00 ± 0.00 ^e^

Results are expressed as the mean ± SD (*n* = 6). For each extract, different superscript lowercase letters indicate statistically different means within each column (*p* < 0.05). MIC—minimum inhibitory concentration and MFC—minimum fungicidal concentration.

**Table 6:** Interactions between TaEO and synthetic fungicides against *Foa*.

Fungicides (SF)	Sample	FIC_SF_	FIC_TaEO_	FICI	Interpretation
SAAF 75%	TaEO	0.5	0.15	0.65	Additive
Carbendazim 50%	TaEO	0.25	0.15	0.4	Synergistic

**Table 7:** IC_50_ (mg/mL) values of antioxidant activity of *T. annuum* crude extracts.

Assay		IC_50_ Values of Crude Extracts (mg/mL)		
		Stems	Leaves	Flowers
DPPH radical-scavenging activity	TaME	0.55 ± 0.02 ^Ab^	0.65 ± 0.02 ^Aa^	0.63 ± 0.01 ^Aa^
	TaHME	0.22 ± 0.01 ^Bb^	0.54 ± 0.02 ^Ba^	0.55 ± 0.03 ^Ba^

Results are expressed as the mean ± SD for three replicates. For each part and extract, different superscript uppercase and lowercase letters indicate statistically different means within each column and row (*p* < 0.05), respectively. Ascorbic acid (commercial standard) was used as a positive control (IC_50_ = 0.036 ± 0.002 mg/mL).

## Data Availability

All data are contained within the article.
